# Modulation of the lung inflammatory response to ozone by the estrous cycle

**DOI:** 10.14814/phy2.14026

**Published:** 2019-03-07

**Authors:** Nathalie Fuentes, Noe Cabello, Marvin Nicoleau, Zissis C. Chroneos, Patricia Silveyra

**Affiliations:** ^1^ Department of Pediatrics The Pennsylvania State University College of Medicine Hershey Pennsylvania; ^2^ Biobehavioral Laboratory The University of North Carolina at Chapel Hill Chapel Hill North Carolina

**Keywords:** Air pollution, follicular phase, inflammation, luteal phase, sex hormones

## Abstract

Emerging evidence suggests that sex differences exist in the control of lung innate immunity; however, the specific roles of sex hormones in the inflammatory response, and the mechanisms involved are unclear. Here, we investigated whether fluctuations in circulating hormone levels occurring in the mouse estrous cycle could affect the inflammatory response to air pollution exposure. For this, we exposed female mice (C57BL/6J, 8 weeks old) at different phases of the estrous cycle to 2 ppm of ozone or filtered air (FA) for 3 h. Following exposure, we collected lung tissue and bronchoalveolar lavage fluid (BAL), and performed lung function measurements to evaluate inflammatory responses and respiratory mechanics. We found a differential inflammatory response to ozone in females exposed in the luteal phase (metestrus, diestrus) versus the follicular phase (proestrus, estrus). Females exposed to ozone in the follicular phase had significantly higher expression of inflammatory genes, including Ccl2, Cxcl2, Ccl20, and Il6, compared to females exposed in the luteal phase (*P *<* *0.05), and displayed differential activation of regulatory pathways. Exposure to ozone in the follicular phase also resulted in higher BAL neutrophilia, lipocalin levels, and airway resistance than exposure in the luteal phase (*P* < 0.05). Together, these results show that the effects of ozone exposure in the female lung are affected by the estrous cycle phase, and potentially hormonal status. Future studies investigating air pollution effects and inflammation in women should consider the menstrual cycle phase and/or circulating hormone levels.

## Introduction

The clinical course of inflammatory lung diseases is influenced by several factors including sex, hormones, and poor air quality (Doherty et al. 2009; Liptzin et al. 2015; Silva et al. 2017). Sex differences in lung disease incidence, prevalence, and severity have been noted for years, as well as increased prevalence rates for asthma, chronic obstructive pulmonary disease (COPD), and other inflammatory diseases in women versus men (Fuseini and Newcomb 2017; Tsiligianni et al. 2017; Center for Disease Control and Prevention, 2018; Shah and Newcomb 2018). The number of women diagnosed with asthma, bronchitis, emphysema, COPD, pneumonia, and lung cancer has significantly increased in the past decade at higher rates than for men, and the mortality associated with many of these conditions is also now higher for women than men (Martinez et al. 2007; Aryal et al. 2014; Jenkins et al. 2017). The current prevalence of asthma in adult women (10.4%) is almost double than that of men (6.2%) in the United States (US) (Center for Disease Control and Prevention, 2018). Women are also 40% more likely to develop COPD than men and account for more than half of all COPD deaths in the US (Ferrari et al. 2010). Accordingly, the risk of an asthma exacerbation requiring hospital admission is twice as high for women than for men (Hyndman et al. 1994; Johnston and Sears 2006). These sex differences, together with the observed crossover in the incidence of asthma versus age, where asthma trends move from higher rates in boys than girls before puberty to higher rates in women than men of reproductive age, suggest that hormonal factors may influence lung inflammatory responses to the environment (Postma 2007). However, little is known about the mechanisms behind these sex differences, and treatments for lung disease continue to be the same for male and female patients.

Air pollution exposure can trigger serious inflammatory responses in susceptible individuals. In patients with lung disease, particularly women, exposure to air pollutants has been associated with higher exacerbation and hospitalization rates and increased morbidity and mortality (Guarnieri and Balmes 2014; Kurt et al. 2016). Ground‐level ozone, one of the dominant air pollutants, is a reactive oxidant gas that can affect lung immunity and exacerbate symptoms in patients with lung disease (Hernandez et al. 2018). Numerous studies have shown that there is a link between short‐term ozone exposure and the incidence of asthma, idiopathic pulmonary fibrosis, and COPD, as well as dose‐dependent declines in spirometric measurements of lung function and increases in airway resistance, indicating that ozone is a potent toxicant for the respiratory system (Tager et al. 2005; Ciencewicki et al. 2008; Fernandez et al. 2015; Sesé et al. 2018).

Investigators have reported variations in lung disease prevalence and respiratory infection at different moments of women's reproductive life (Skobeloff et al. 1996). For example, many women experience lung disease exacerbations throughout the luteal and follicular phases of the menstrual cycle, as well as during pregnancy, oral contraceptive use, and menopause, indicating that female sex hormones can modulate the inflammatory response (Pauli et al. 1989; Chhabra 2005; Tam et al. 2011; Rao et al. 2013; Matteis et al. 2014). A few studies in cells and animal models have also shown that sex hormones can affect airway tone, inflammation, immune cell function, and transcription of specific genes that alter host immunity and promote immune cell proliferation (Chang and Mitzner 2007; Fuseini and Newcomb 2017; Yung et al. 2018). While studies in cells and animal models have addressed the contributions of male and female sex hormones to lung immunity (Keselman and Heller 2015; Keselman et al. 2017; Fuseini et al. 2018), only a few studies have addressed the mechanisms by which physiological hormonal fluctuations that occur in the female reproductive cycle affect the inflammatory response (Fuentes et al. 2018; Fuentes and Silveyra 2019).

Regarding ozone exposure, human studies exploring sex differences in susceptibility, and the influence of the female menstrual cycle have provided conflictive and inconclusive results (Fox et al. 1993; Weinmann et al. 1995; Seal et al. 1996; Medina‐Ramón and Schwartz 2008; Bell et al. 2014; Vinikoor‐Imler et al. 2014). Furthermore, recent sex‐specific disaggregation of data in the 2017 Global Burden of Diseases (GBD) study uncovered areas where these aspects can be further explored and acted upon, and showed that substantial differences between men and women that underlie the overall headline figures are still pervasive and too often overlooked (Collaborators, Global Burden of Disease, 2018; Lancet 2018). Overall, this suggests that more research on lung disease mechanisms is needed for the development of more personalized preventative and treatment options for men and women that would consider the patient's sex and hormonal status.

To study hormonal regulation of physiological functions, researchers have used animal models involving the estrous cycle (Silveyra et al. 2007b; Yip et al. 2013). In the mouse, the estrous cycle, which repeats every 4–5 days, has two phases comprised of four stages: follicular (proestrus, estrus), and luteal (metestrus, diestrus) (Byers et al. 2012). In the follicular phase, corpora lutea regress and progesterone levels decline, while preovulatory follicles grow and estrogen levels increase. A peak of luteinizing hormone (LH) precedes ovulation in the evening of proestrus, leading to estrus. Following ovulation, the corpora lutea develop throughout metestrus and diestrus, when progesterone levels increase. Therefore, the physiological variations of hormone levels occurring during the cycle represent a powerful tool to study the effects of sex hormones in many organs (i.e., the lung). By investigating females in a state of high or low levels of ovarian hormones related to the estrous cycle, researchers have demonstrated the existence of estrous cycle‐specific sex differences in tissues such as the brain and lung (Duclot and Kabbaj 2015; Fuentes et al. 2018; Fuentes and Silveyra 2019).

Previously, we reported sex differences in both gene expression and miRNA signatures in response to ozone exposure using mouse models (Cabello et al. 2015; Fuentes et al. 2018). Furthermore, we reported differences in signaling cascades activated in female mice exposed to ozone in different estrous cycle stages (Mishra et al. 2016). Based on these data, we hypothesized that the stage of the estrous cycle, and more specifically the circulating levels of female hormones in each cycle phase can influence the inflammatory response to ozone. To test this hypothesis in this study, we exposed female mice to ozone or filtered air (FA) at different stages of the estrous cycle, and then compared the overall inflammatory response among groups by analyzing lung cell profiles and measuring expression of inflammatory genes. We found that the phase of the estrous cycle (luteal vs. follicular) strongly affects the response to ozone. Together, the results we present here indicate that circulating female hormones can regulate inflammatory processes in the lung, and suggest that future studies should consider the estrous cycle phase when evaluating lung inflammatory outcomes in females.

## Materials and Methods

### Animals

We obtained adult female C57BL/6J mice (8 weeks old) from JAX laboratories (Bar Harbor, ME) and housed in a 12/12‐h light/dark cycle (6:00 am–6:00 pm) with food and water ad libitum. The Penn State College of Medicine Institutional Animal Care and Use Committee (IACUC) approved all procedures.

### Estrous cycle

Estrous cycle stages were assessed daily for a minimum of 2 weeks prior to exposure experiments. As described by us in the past, cycle stages were determined by the amount of nucleated epithelial cells, leukocytes, and cornified cells in vaginal smears following traditional protocols (Caligioni 2009; Mishra et al. 2016; Fuentes and Silveyra 2019). In addition, stages were confirmed by serum determinations of estradiol, LH, and progesterone via ELISA (cats. #MBS9424676, #MBS266675 and #MBS041300, MyBioSource, San Diego, CA) in samples obtained at 6:00 pm (4 h postexposure). Females with three consecutive cycles of 4–5 days in length were considered regular (Ekambaram et al. 2017). Mice with irregular cycles were excluded from the experiment.

### Ozone exposure

**Figure 1:**
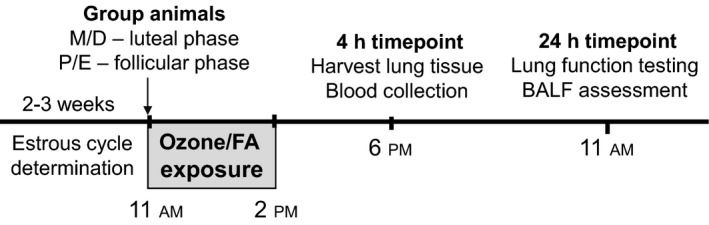
Study design. Estrous cycle stages were assessed daily for a minimum of 2 weeks prior to ozone exposure experiments. Female mice at different stages of the estrous cycle were exposed to 2 ppm of ozone or filtered air (FA, control) for 3 h between 11:00 am and 2:00 pm (*n *= 6–8 per phase/exposure group). Four hours after exposure (6:00 pm), blood and lung tissue samples were harvested. Lung function testing and BALF collection were performed at 24 h postexposure (11:00 am).

**Figure 2:**
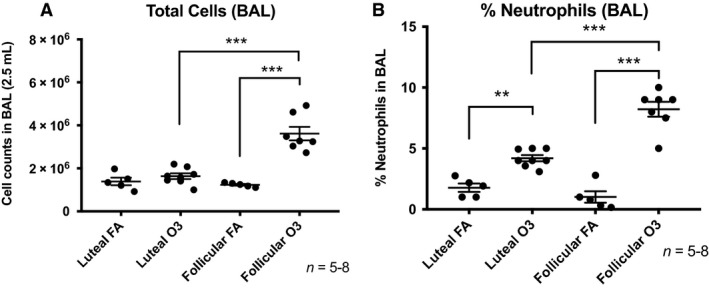
(A) Bronchoalveolar lavage (BAL) fluid cell counts measured at 24 h postexposure in female mice exposed for 3 h to 2 ppm ozone or filtered air in the luteal or follicular phase of the estrous cycle. (B) Total BAL (2.5 mL) cells and polymorphonuclear neutrophils (% of total) measured at 24 postexposure to ozone or filtered air. Results are expressed as means ± SEM of 5–8 mice per group (**P < 0.01, ***P < 0.001). FA: filtered air, O3: ozone.

We exposed female mice at different stages of the estrous cycle (Fig. 1) to 2 ppm of ozone or FA (control) for 3 h (*n* = 6–8 per phase/exposure group) using an exposure chamber as described in (Cabello et al. 2015). The system delivers a controlled air flow (> 30 air changes/h) with regulated temperature (25°C) and relative humidity (50%). Animals were exposed between 11:00 am and 2:00 pm to account for circadian fluctuations of circulating hormone levels (Fig. 1). Four hours after exposure, blood was collected for serum determinations, and lung tissue samples were harvested in liquid nitrogen, pulverized, and stored at −80°C until use.

### RNA preparation

We extracted total RNA from pulverized lung tissue using the Direct‐Zol kit (Zymo Research, Irvine, CA). RNA concentration and quality were analyzed by Nanodrop and Bioanalyzer (RIN  > 7) at the Pennsylvania State University College of Medicine Genome Sciences Core Facility.

### mRNA arrays

Purified RNA (400 ng) was retro‐transcribed using the RT^2^ First Strand Kit (QIAGEN, Germantown, MD), and amplified using the Mouse Inflammatory Response and Autoimmunity PCR Array PAMM‐077Z (QIAGEN, Germantown, MD) in the QuantStudio 12K Flex system (Life Technologies, Carlsbad, CA). A list of genes in this array can be viewed at: https://www.qiagen.com/us/shop/pcr/primer-sets/rt2-profiler-pcr-arrays/?catno=PAMM-077Z. Results (Ct values) were extracted with the QuantStudio 12K Flex Software, and exported to MS excel. Ct values for each sample were then normalized to the average Ct of four endogenous controls (*β‐actin*,* β2‐microglobulin*,* GAPDH*, and *Hsp90*) as ΔCt = (Ct_Target − Ct_housekeeping), and fold changes were calculated using the 2^−ΔΔCT^ method (Livak and Schmittgen 2001), as described by us previously. For fold change calculations, ΔΔCt based fold change values were obtained using a control sample with the 2^−ΔΔCT^ formula, where –ΔΔCt = −[ΔCttest − ΔCtcontrol]. We generated heatmaps of fold changes calculated with this method using the R software, and we submitted datasets to the Gene Expression Omnibus (https://www.ncbi.nlm.nih.gov/geo/query/acc.cgi?acc=GSE123276) and our laboratory repository: http://psilveyra.github.io/silveyralab/.

### Real‐time PCR

For selected validation experiments, we retro‐transcribed 600 ng of DNA‐free RNA from a separate set of mice using the High Capacity cDNA reverse transcription kit (Thermo Fisher, Waltham, MA). To quantify gene expression, we used selected TaqMan assays (Life Technologies, Carlsbad, CA) to perform PCR amplifications. We used the following probes: Ccl20 (Mm01268754), Il6 (Mm00446190), Cxcl2 (Mm00436450), and Ccl2 (Mm00441242). Results were analyzed using the QuantStudio 12K Flex Software, and normalized to 18s rRNA expression (no. Mm03928990) using the relative quantification method (Livak and Schmittgen 2001).

### Ingenuity pathway analysis (IPA)

Using IPA (Qiagen, Redwood City, CA) on array data, we determined gene interaction networks, top diseases, and molecular functions based on prediction scores, as described in (Cabello et al. 2015).

### Lung function determinations and BAL analysis

At 24 h postexposure (Fig. 1), we anesthetized mice with ketamine, (130 mg/kg), xylazine (10 mg/kg), and vecuronium bromide (1 mg/kg) i.p., and connected them to a flexiVent system (SCIREQ Inc, Canada) at a respiratory rate of 150 breaths/min and a positive end‐expiratory pressure of 3 cm H_2_O. We obtained respiratory mechanics parameters following administration of 0–50 mg/mL of methacholine (Sigma‐Aldrich, St Louis, MO), as described in Fino et al. (2017). We recovered BAL cells in 2.5 mL of DPBS with 1 mmol/L EDTA and analyzed as described in Cabello et al. (2015). We determined lipocalin‐2/NGAL levels by ELISA (R&D Systems, Minneapolis MN, kit #MLCN20) in 50 *μ*L of BAL.

### Data analysis

For gene arrays, we performed statistical analyses in R using the Bioconductor *limma* package, and defined differential expression as a Benjamini–Hochberg False Discovery Rate (FDR) < 0.05 (Benjamini and Hochberg 1995). For gene expression, cell counts, and lipocalin, we analyzed differences by one‐way ANOVA followed by Tukey's post hoc test (alpha = 0.05).

## Results

### Confirmation of estrous cycle stages

After determining the estrous cycle stages by vaginal smears, we confirmed them by serum levels of estradiol, progesterone, and LH at 6:00 pm (4 h after exposure to ozone or FA). As expected, we found predominantly higher levels of estradiol at 6:00 pm in the proestrus stage, and of progesterone in diestrus (Table 1). We observed the preovulatory peak of LH in proestrous females. For analysis of inflammation, lung function, and gene expression outcomes, we grouped animals by follicular (proestrus/estrus) or luteal (metestrus/diestrus) phases.

**Table 1:** Serum hormone levels measured at 6:00 pm in female mice at different stages/phases of the estrous cycle

Estrous cycle phase	Luteal		Follicular	
Estrous cycle stage	Metestrus	Diestrus	Proestrus	Estrus
LH (ng/mL)	5.23 ± 1.43	5.02 ± 0.81	11.1 ± 3.21	5.83 ± 2.13
Estradiol (pg/mL)	3.80 ± 0.35	2.59 ± 0.36	7.75 ± 0.55	3.74 ± 0.39
Progesterone (ng/mL)	2.15 ± 0.31	9.09 ± 3.01	2.98 ± 0.77	2.60 ± 0.56

### The estrous cycle phase affects the inflammatory response to ozone

**Figure 3:**
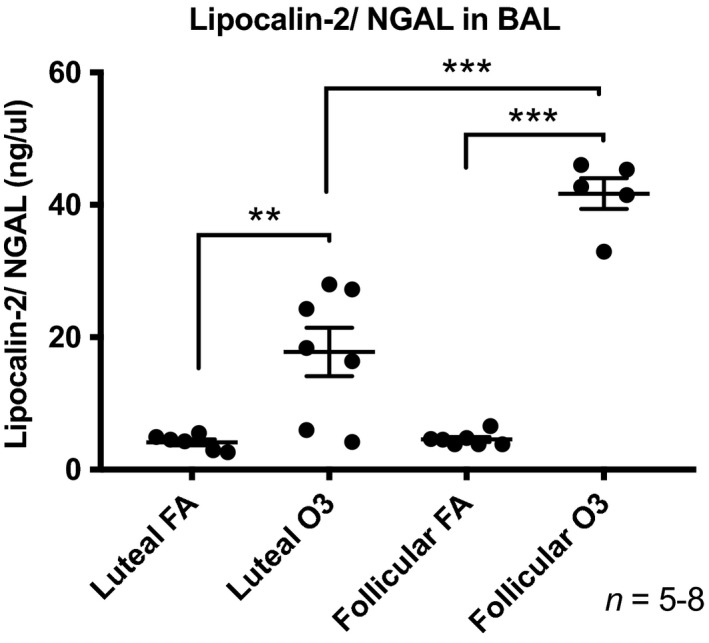
Lipocalin‐2 (NGAL) levels measured by ELISA in BAL collected at 24 h postexposure from female mice exposed to ozone (2 ppm) or filtered air for 3 h in the luteal or follicular phase of the estrous cycle. Results are expressed as means ± SEM of 5–8 mice per group (**P < 0.01, ***P < 0.001). FA: filtered air, O3: ozone.

As expected, exposure to ozone resulted in increased cell numbers and neutrophilia in BAL fluid obtained at 24 h postexposure (Fig. 2). Interestingly, when comparing BAL differential cell counts from mice exposed to ozone in the follicular versus the luteal phase, we found that mice in the follicular phase displayed significantly higher total cell and neutrophil percentage than females exposed to ozone in the luteal phase (Fig. 2) (2.2‐fold and twofold, respectively, *P *<* *0.05). We observed no significant changes in macrophage and lymphocyte counts across groups (data not shown). In addition, the levels of the lung injury marker neutrophil gelatinase‐associated lipocalin (lipocalin‐2/NGAL) were also significantly higher in BAL fluid of females exposed to ozone in the follicular phase versus the luteal phase (Fig. 3). There were no differences in lipocalin levels in BAL of mice exposed to FA in different estrous cycle phases.

### Exposure to ozone in females at different phases of the estrous cycle induces differential gene expression signatures

**Figure 4:**
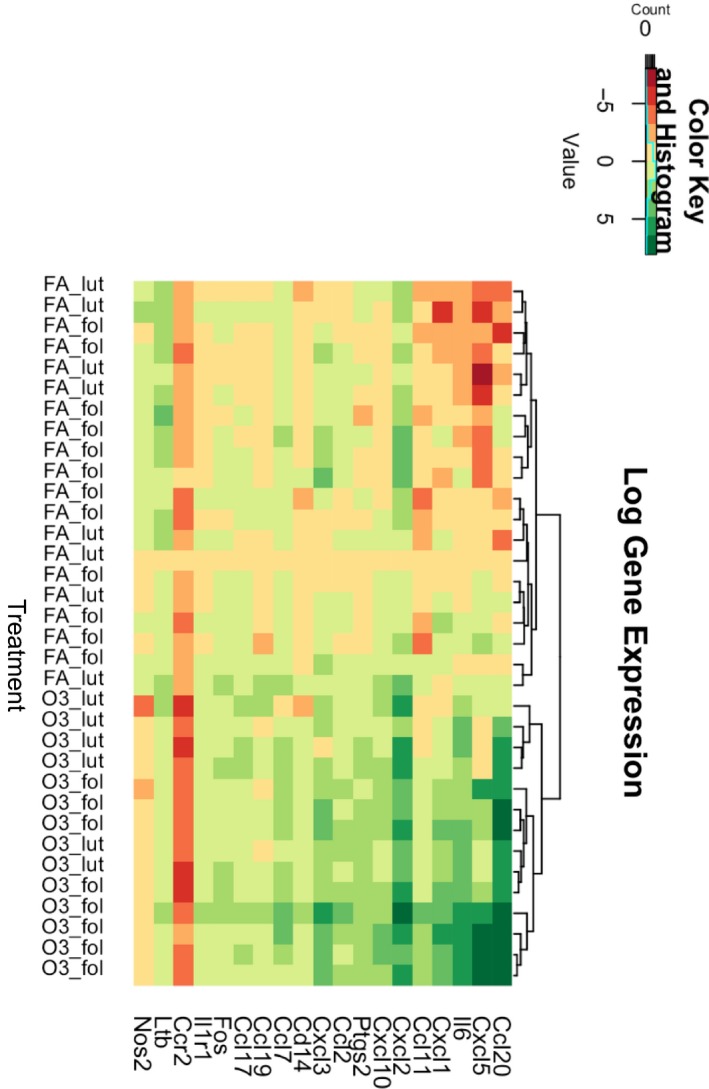
Heatmap (cluster analysis of log gene expression) of top differentially expressed genes measured by a PCR array in lung tissue collected at 4 h postexposure from female mice exposed to ozone (2 ppm) versus filtered air in the luteal and follicular estrous cycle phases (*n* = 6–8). FA, filtered air; O3, ozone; fol, follicular phase; lut, luteal phase.

**Figure 5:**
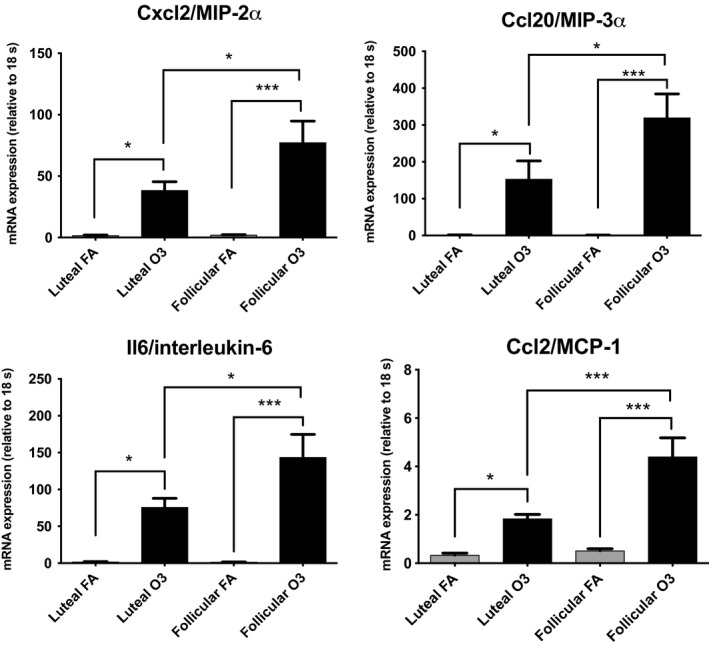
Relative mRNA expression of selected genes (Ccl20, Cxcl2, Ccl2, Il6) analyzed by real‐time PCR in lung tissue collected at 4 h postexposure from female mice exposed to ozone (O3) or filtered air (FA) in the luteal or follicular phase of the estrous cycle (*n* = 12). Results are expressed as means ± SEM. Significant differences were analyzed by ANOVA (*P < 0.05, ***P < 0.001).

To test whether the estrous cycle phase affected the inflammatory response to ozone, we screened for the expression of 84 inflammatory genes in total lung homogenates from mice exposed to ozone or FA in the follicular or luteal phase using a PCR array. Gene expression array results revealed three genes with significantly higher expression in mice exposed to ozone versus FA in the luteal phase: Ptgs2 (log fold change = 2.692); Ccl17 (log fold change =  2.204); Myd88 (log fold change = 2.82) (Table 2). In contrast, in females exposed to ozone in the follicular phase, the expression of 16 genes (14 upregulated, 2 downregulated) was significantly altered when compared to mice exposed to FA (Table 3). Most of the genes induced in this phase were inflammatory chemokines including Ccl20 (log fold change = 105.174), Cxcl5 (log fold change = 81.891), Cxcl2 (log fold change = 37.364), and Cxcl1 (log fold change = 13.174), as well as cytokines such as Il6 (log fold change = 28.000) (Table 3). A representative heatmap of array results including a cluster analysis is shown in Figure 4. When we verified expression of some of these differentially expressed genes using real‐time PCR, we found that even though exposure to ozone resulted in significantly higher expression of inflammatory cytokines regardless of the estrous cycle phase, the levels of Ccl20, Cxcl2, Il6, and Ccl2 where significantly higher in lungs of females exposed to ozone in the follicular phase when compared to the luteal phase (Fig. 5). Together, these results indicate that the hormonal milieu associated with the estrous cycle phase could influence gene expression mechanisms activated in response to ozone challenge.

**Table 2:** Differential inflammatory gene expression in lung tissue from females exposed to ozone versus filtered air in the luteal phase of the estrous cycle

Gene symbol	logFC	FDR
Myd88	2.820	0.024
Ptgs2	2.692	0.020
Ccl17	2.204	0.020

**Table 3:** Differential inflammatory gene expression in lung tissue from females exposed to ozone versus filtered air in the follicular phase of the estrous cycle

Gene symbol	logFC	FDR
Ccl20	105.174	1.12E‐08
Cxcl5	81.891	5.20E‐07
Cxcl2	37.364	9.30E‐07
Il6	28.000	9.89E‐07
Cxcl1	13.174	9.69E‐06
Cxcl10	5.010	4.43E‐05
Ccl2	4.199	4.55E‐04
Ccl7	3.856	4.55E‐04
Ptgs2	3.827	4.55E‐04
Ccl11	3.389	4.55E‐04
Cd14	1.796	5.12E‐03
Ccl17	1.768	5.12E‐03
Fos	1.704	8.11E‐03
Il1r1	0.782	8.11E‐03
Nos2	−0.966	8.11E‐03
Ltb	−2.238	1.71E‐02

### IPA reveals estrous cycle‐dependent activation of gene networks

**Figure 6:**
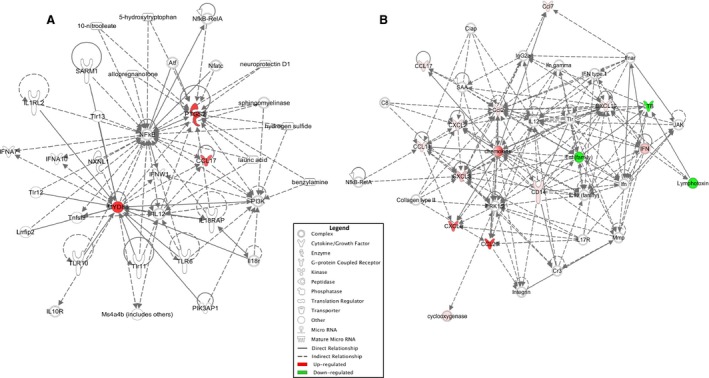
Comparison of networks affected by filtered air or ozone exposure in females exposed at different phases of the estrous cycle. (A) Diagram of biological networks of regulatory pathways whose expression were up‐ and downregulated in the lungs of animals exposed to filtered air (FA) versus ozone in the luteal phase. (B) Network analysis from animals exposed to FA versus ozone in the follicular phase. Both diagrams show reported direct (solid lines) and indirect (dashed lines) interactions. Molecules that are downregulated or upregulated are represented as a node in green or red, respectively.

IPA revealed that the top molecular functions associated with differentially expressed genes in female mice exposed to ozone versus FA during the follicular phase were cell‐to‐cell signaling and interaction, cellular movement, and immune trafficking (Table 4). These genes are related to important regulators of the immune system such as interleukin 17 receptor (IL‐17R) and janus kinases (JAK), which may be altering the lung host defense. For differentially expressed genes in the luteal phase, IPA showed that these were linked to cellular function and maintenance, and cellular growth and proliferation, which are important pathways in the lung inflammatory response (Fig. 6). Moreover, the top associated network functions with these genes included inflammatory response, infectious diseases, and organismal injury (Table 5). Several genes were also associated with key regulators of the immune response including toll‐like receptors (TLRs), interferons (IFNs), and the transcription factor NFkB.

**Table 4:** Target genes and associated pathways for differentially expressed genes in lung tissue of female exposed to ozone mice in the follicular phase

A. Molecules associated with differentially expressed genes					
C8	IFN‐I	CCL11	TLR	IFNB	IFN*γ*
CCL20	IL12	CCL7	CCL17	MMP	IL12
Ciap	LTB	CXCL3	CD14	TNF	JAK
CXCL2	SAA	IFNAR	CR3	CCL2	NF*κ*B
ERK1/2	CCL11	IL17R	CXCL6	CXCL10	
B. Differences in top diseases and bio‐functions					
Diseases and disorders				*P* Value	
Connective tissue disorders				1.78E‐04–1.84E‐16	
Immunological disease				2.48E‐04–1.84E‐16	
Inflammatory disease				1.78E‐04–1.84E‐16	
C. Top molecular and cellular functions					
Molecular and cellular functions				*P* Value	
Cell‐to‐cell signaling and interaction				2.91E‐04–1.08E‐18	
Cellular movement				2.97E‐04–1.08E‐18	
Cellular development				2.61E‐04–6.48E‐12	
D. Top physiological system development and function					
Development and function				*P* Value	
Immune cell trafficking				2.62E‐04–6.08E‐18	
Hematological system development and function				2.89E‐04–5.41E‐17	
Tissue development				2.61E‐04–2.61E‐15	
E. Top associated network functions					
Associated network functions				Score	
Cell‐to‐cell signaling and interaction, cellular movement, immune cell trafficking				31	

**Table 5:** Target genes and associated pathways for differentially expressed genes in lung tissue of female exposed to ozone mice in the luteal phase

A. Molecules associated with differentially expressed genes					
IFNA7	CCL17	TLR10	PTGS2	IL12	TLR13
IL18R	IFNW1	TLR6	TLR11	NXNL1	PIK3AP1
NFATC	IL18RAP	IL10R	TNF	SARM1	MYD88
PI3K	NFKB	ILIRL2	ATF	TLR12	IFNA10
B. Differences in top diseases and bio‐functions					
Diseases and disorders				*P* Value	
Cancer				1.96E‐03–1.20E‐06	
Gastrointestinal disease				1.96E‐03–1.20E‐06	
Hereditary disorder				5.62E‐04–1.20E‐06	
C. Top molecular and cellular functions					
Molecular and cellular functions				*P* value	
Cellular function and maintenance				1.68E‐03–1.31E‐08	
Cellular growth and proliferation				1.82E‐03–7.89E‐08	
Cellular movement				1.40E‐03–2.58E‐06	
D. Top physiological system development and function					
Development and function				*P* value	
Tissue development				1.96E‐03–1.31E‐08	
Tissue morphology				1.68E‐03–1.31E‐08	
Cell‐mediated immune response				1.66E‐03–2.58E‐06	
E. Top associated network functions					
Associated network functions				Score	
Inflammatory response, infectious diseases, organismal injury, and abnormalities				9	

### Exposure to ozone increases airway resistance in females in the follicular phase but not the luteal phase

**Figure 7:**
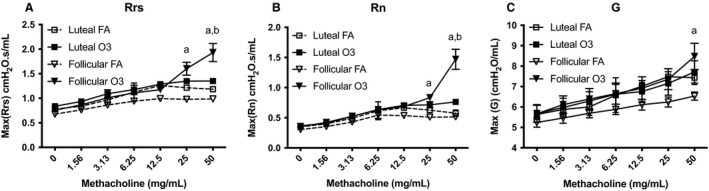
Effects of ozone exposure on lung mechanical properties in female mice at different phases of the estrous cycle. (A) Respiratory system resistance (Rrs). (B) Newtonian resistance (Rn). (C) Tissue damping (G). Respiratory function parameters were measured with a flexiVent system in female mice at 24 h after exposure to ozone (solid line) or filtered air (dashed line) in the follicular (inverted triangle) or luteal (square) phase of the estrous cycle. Exposure to ozone significantly increased Rrs and Rn at higher doses of methacholine in mice the follicular phase but not the luteal phase. Results are expressed as mean ± SEM of data from 4 to 6 mice per group. FA, filtered air; O3, ozone; ^a^significantly different than luteal FA (*P* < 0.05), ^b^significantly different than luteal O3 (*P* < 0.05).

To determine whether ozone affects lung function differentially across the estrous cycle, we compared lung function parameters in mice exposed in the follicular or luteal phase. Our results show that female mice exposed to ozone in the follicular phase displayed significantly higher maximum respiratory system resistance (Rrs) and maximum Newtonian resistance (Rn) at higher doses of methacholine than females exposed to ozone in the luteal phase (Fig. 7). Also, female mice exposed to ozone in the follicular phase showed a slight increase in tissue damping (G), a parameter related to tissue resistance in small airways, when compared to mice exposed to ozone in the luteal phase (Fig. 7). We did not observe any changes in airway resistance in mice exposed to FA.

## Discussion

Sex differences have been reported in both the severity of inflammatory lung diseases and susceptibility to environmental challenges, but the mechanisms associated with these disparities have not yet been clearly defined. In our previous work, we identified a sexually dimorphic response to ozone challenge, manifested by sex differences in airway inflammation and the expression of lung inflammatory mediators and miRNAs (Cabello et al. 2015; Fuentes et al. 2018). Here, we expanded our investigations to the study of the female lung, and specifically the influence of circulating sex hormone levels as well as other physiological changes occurring throughout the estrous cycle in lung inflammation. Our results suggest that the inflammatory response to acute ozone exposure is strongly dependent on the estrous cycle phase in female mice. We found differences in activation of gene expression networks, immune cell profiles, and changes in lung mechanics when comparing mice exposed to ozone in the estrous cycle follicular versus luteal phases. To our knowledge, this study is the first to identify differences in the inflammatory response and lung function changes associated with ozone exposure in females across the estrous cycle.

Throughout the female reproductive cycle, the lung is exposed to physiological variations in circulating sex hormone levels. Human menstrual cycles (28 days) and rodent estrous cycles (4–5 days) share similar patterns of serum hormone levels and undergo follicular and luteal phases (Sato et al. 2016). In addition, lung cells also express sex hormone receptors, including estrogen and progesterone nuclear and membrane receptors, known to mediate several functions of the organ, and specifically immune responses (Fuentes and Silveyra 2018). Some studies, including ours, have hypothesized that female sex hormones can act as functional regulators of lung function and immunity through regulation of inflammatory gene expression (Card and Zeldin 2009; Tam et al. 2011; Cabello et al. 2015). In the current study, we sought to study the influence of the preovulatory estrogen surge occurring in the afternoon of proestrus on the expression of inflammatory genes in lung tissue. We achieved this by exposing mice in proestrus and estrus (i.e., follicular phase) to ozone or FA, and comparing the response to that of mice exposed in metestrus and diestrus (i.e., luteal phase). By using this approach, we could assess the influence of the estrous cycle hormonal milieu, including fluctuations in estrogen and progesterone levels, in ozone‐induced inflammation. Our approach identified estrous cycle phase specific gene expression signatures revealing higher proportion of genes differentially expressed in the follicular phase (16 genes) versus the luteal phase (three genes), as well as differences in predicted inflammatory pathways and gene networks activated upon ozone exposure. Together, these results suggest that the rising levels of estrogen and/or the declining levels of progesterone in the follicular phase may play a role in the regulation of inflammatory gene transcription. Future studies investigating estrogen and/or progesterone receptor regulation of gene expression in the lung may elucidate the mechanisms involved in the observed differences.

Our results show that females exposed to ozone in the follicular phase displayed a vast expression of chemotactic factors and lung injury markers. This is of particular interest because chemoattractants such as Cxcl2, Ccl20, and Ccl2 are known to induce directed migration of immune cells to specific sites (e.g., the lung), and may explain the increased neutrophilia observed in mice exposed to ozone in this phase versus the luteal phase. We also found upregulation of Myd88 in mice exposed to ozone during the luteal phase. This is an interesting finding, because variations in Myd88 have been shown to alter toll‐like receptors expression (TLR6, TLR10, TLR11, and TLR13), which are associated with expression of inflammatory mediators such as NFkB, IFNs, TNFs, IL‐12, and IL‐18. In addition, levels of lipocalin‐2/NGAL were significantly higher in mice exposed to ozone in the follicular phase when compared to all the other groups. Lipocalin‐2/NGAL is secreted mostly by neutrophils during lung inflammation or injury (Xiao and Chen 2017). Therefore, upregulation of lipocalin‐2/NGAL levels in the follicular phase correlates with the increase in the percentage of neutrophils seen. Overall, when comparing inflammatory pathways, the follicular phase is associated with cell‐to‐cell signaling and interaction, cellular movement, immune cell trafficking, and lung injury biomarkers, while the luteal phase is characterized by inflammatory response, infectious diseases, organismal injury, and abnormalities.

Our findings in this mouse model have several clinical implications. Epidemiological studies in women have shown variations in lung function and asthma exacerbations throughout the menstrual cycle. Some women report worsening of symptoms in the periovulatory period, and with most complications occurring in the pre‐ or peri‐menstrual period (Zein and Erzurum 2015; DeBoer et al. 2018). Therefore, it is likely that asthmatic women exposed to high ozone levels during the follicular phase experience a worsening of symptoms versus asthmatic women exposed in the luteal phase. Alternatively, this could also apply to women with other inflammatory diseases, as well as healthy women. We believe clinical studies assessing the effects of environmental exposures in women should consider this variable when interpreting results (Fox et al. 1993).

When we compared lung mechanics in mice exposed to ozone in the follicular and luteal phases, we found that female mice exposed to ozone in the follicular phase exhibited significantly higher airway resistance at higher doses of methacholine than females exposed to ozone in the luteal phase. These findings were consistent with a previous study showing differences in airflow capacity in the luteal phase versus follicular phase in women with asthma (Farha et al. 2009). One explanation for these results is that inhaled ozone reacts with substrates including lipids and proteins in the lung lining fluid, initiating a number of cellular responses in the lung epithelium (Mudway and Kelly 2004). The resulting production of cytokines and chemokines and increased expression of cell surface molecules can influence activation of immune cells in the pulmonary microvasculature and their extravasation into the interstitial space and airways. It is likely that these reactive byproducts can directly damage lung compartments and/or increase mucus production, resulting in the observed changes in airway physiology and increased lung resistance. Moreover, studies have shown that estrogen can also upregulate mucus production in airway epithelial cells, and that progesterone can decrease contractility and increase relaxation of bronchial smooth muscle cells, thus contributing to the observed differences in lung function parameters of females exposed to ozone in the luteal versus the follicular phase (Sathish et al. 2015).

While our results support the hypothesis that circulating female hormone levels in each cycle phase can influence the inflammatory response to ozone, our study has several limitations. One limitation is the grouping of estrous cycle stages into follicular and luteal phases. It is likely that specific hormonal or nonhormonal changes associated with each estrous cycle stage could influence the inflammatory response to ozone. Future studies involving higher number of animals are likely to address this limitation. Such studies should also consider circadian variations in gonadal and pituitary hormone levels in the rodent (Silveyra et al. 2007a, 2009). A second limitation of our model is the use of whole lung tissue to study gene expression differences. As conducted, our approach cannot identify changes in specific cell types, such as resident or recruited immune cells, as well as in lung compartments such as the endothelium or vascular, which have been identified as important contributors of sexual dimorphism in lung diseases such as asthma (Prakash 2016). In fact, some of the differential gene expression networks identified display associations with connective tissue disorders, cell‐to‐cell signaling, and hematologic systems. In addition, the gene expression array used was selected to assess genes associated with inflammatory and autoimmunity responses. Additional gene expression changes triggered by ozone in the lung may be detected in future studies using high throughput gene expression detection techniques. Finally, this study was conducted in only one mouse strain. This is considered a study limitation, since it is known that the response to acute ozone exposures is strain‐specific in mice (Kleeberger et al. 1993). Future experiments assessing hormonal influences in the response to ozone exposure should consider incorporating additional mouse strains.

In summary, the present study revealed differences in lung inflammatory gene expression, immune cell profiles, and lung function in response to ozone in female mice exposed at different phases of the estrous cycle. Our observations suggest that sex hormones can influence inflammatory processes in the lung, and that the effects of environmental exposures on women's lung health could be affected by hormonal status. Future studies should evaluate the role of specific sex hormones in the control of gene expression and cell signaling in response to ozone in both healthy and diseased lungs. These will help us understand the physiopathology of lung disease triggered by environmental exposures and hormonal status in women, and will likely uncover points of intervention for lung disease therapies that will be specific for women.

## Conflict of Interest

The authors declare that they have no conflict of interest.
